# Macrophage Depletion by Intracerebroventricular Administration of Clodronate-Liposome Attenuates the Development of Angiotensin II-Salt-Induced Neurogenic Hypertension in Rats

**DOI:** 10.31662/jmaj.2025-0203

**Published:** 2025-12-19

**Authors:** Yoshitsugu Sunagawa, Masanobu Yamazato, Yoriko Yamazato, Akio Ishida, Takuto Nakamura, Yusuke Ohya

**Affiliations:** 1 Department of Cardiovascular Medicine, Nephrology and Neurology, Graduate School of Medicine, University of the Ryukyus, Ginowan, Japan; 2 Naha City Public Health Center, Naha, Japan; 3 Okinawa Health Promotion Foundation, Haebaru, Japan; 4 Omoromachi Medical Center, Naha, Japan; 5 Department of Sports and Health Sciences, Faculty of Human Health Sciences, Meio University, Nago, Japan; 6 Nishizaki Hospital, Itoman, Japan; 7 Okinawa Hokubu Medical Foundation, Nago, Japan

**Keywords:** angiotensin II, blood pressure, intracerebroventricular injection, perivascular macrophage, sympathetic nervous system

## Abstract

**Introduction:**

The increase in peripheral angiotensin II level results in neurogenic hypertension with brain inflammation. Macrophages in the cerebrospinal fluid (CSF) influence neuroinflammation through communication between the peripheral and central nervous systems. However, the role of macrophages in neurogenic hypertension development remains unclear. We hypothesized that macrophages in the CSF have a role in the development of angiotensin II-initiated neurogenic hypertension.

**Methods:**

Sprague-Dawley rats with radio-telemetry pressure transducers underwent surgery for the subcutaneous implantation of either saline- or angiotensin II-filled osmotic minipump. They received an intracerebroventricular injection of either phosphate-buffered saline-liposome as a control or clodronate-liposome to deplete macrophages. Postoperatively, rats received 2% salt diet for 14 days. Different groups of rats underwent a hexamethonium challenge test at 7-9 days after treatment initiation to evaluate their sympathetic tone.

**Results:**

Rats with angiotensin II-salt treatment demonstrated a time-dependent arterial pressure increase. Rats receiving angiotensin II-salt treatment with clodronate-liposome had delayed arterial pressure increases and lower mean arterial pressure (91 ± 4 mmHg) than rats receiving control-liposome (111 ± 4 mmHg) on day 8. The angiotensin II-salt treatment increased the peak depressor response to intravenous hexamethonium injection, messenger RNA expression of interleukin-6 and transforming growth factor-ß, and number of Iba1-positive cells in the brainstem. Intracerebroventricular injection of clodronate-liposome attenuated the angiotensin II-salt-induced increases in the depressor response, gene expressions, and cell number.

**Conclusions:**

Our data suggest that macrophages in the CSF are involved in the development of angiotensin II-salt-induced neurogenic hypertension by modulating brain inflammation.

## Introduction

Hypertension is one of the strongest risk factors for cardiovascular diseases, including coronary artery disease, stroke, renal failure, and peripheral arterial disease ^(1)^. Increased sympathetic nerve activity and altered arterial baroreflex function are associated with primary hypertension in humans and various animal models ^(2), (3), (4), (5), (6)^. Inflammation in cardiovascular regulatory areas of the brain is a key mechanism in the development of neurogenic hypertension ^(7)^. Chronic subcutaneous or intravenous administration of angiotensin II induces a slow-pressor effect in normotensive rats and leads to the development of hypertension ^(8), (9)^. Increased sympathetic nerve activity plays an important role in this angiotensin II-induced hypertension. Continuous subcutaneous administration of angiotensin II in normotensive Sprague-Dawley rats leads to increased microglial activation and inflammatory cytokine expression in the paraventricular hypothalamic nucleus. This demonstrates that continuous angiotensin II administration induces inflammation in the cardiovascular regulatory area of the brain ^(10)^. Continuous administration of angiotensin II also increases blood-brain barrier permeability in mice. The angiotensin II type 1a receptor in perivascular macrophages is suggested to be involved in this alteration of blood-brain barrier permeability ^(11)^. This suggests that circulating angiotensin II may reach perivascular macrophages in the cerebrospinal fluid (CSF) and affect blood-brain barrier permeability. Furthermore, CSF macrophages may play a role in spreading inflammatory signals from peripheral blood to the brain parenchyma in animal models of inflammatory brain diseases ^(12), (13)^.

Despite the availability of methods to deplete macrophages in the CSF ^(14), (15), (16), (17), (18)^, studies investigating the role of these macrophages in neurogenic hypertension are lacking. We hypothesized that CSF macrophages may transmit inflammatory signals from peripheral angiotensin II to the brain parenchyma, contributing to sympathetic nerve hyperactivity through the induction of brain inflammation in cardiovascular regulatory areas. We conducted the present study to support or refute our hypothesis.

## Materials and Methods

### Animals

Five-week-old male Sprague-Dawley rats were purchased from Japan SLC Inc. (Hamamatsu City, Shizuoka, Japan) and housed in standard rodent cages with standard laboratory chow and tap water available *ad libitum*. The rats were kept in a room with a constant temperature (24 ± 2°C), humidity (55 ± 10%), and a 12-hour light period from 8 AM to 8 PM. The rats were allowed at least 1 week to acclimate to the environment after their arrival. Animal experiments were approved by the University of the Ryukyus Animal Care and Use Committee (A2017155, A2021011) and conducted in accordance with institutional guidelines.

### Radio-telemetry pressure transducer implantation and blood pressure measurements

Rats were anesthetized by an intraperitoneal injection of 50 mg/kg sodium pentobarbital. Radio-telemetry pressure transducers (Data Sciences International, Arden Hills, MN, USA), consisting of a fluid-filled catheter attached to a PA-C40 transmitter, were implanted into the abdominal aorta as described previously ^(19)^. Before and after surgery, each rat received a prophylactic intramuscular injection of 20,000 U/kg body weight of penicillin G. During the experiments, the rats’ blood pressure (BP) and heart rate (HR) were recorded every 5 minutes for an average of 10 seconds from 9 AM to 12 PM or 1 PM to 4 PM, three times a week. Daily data from each rat were averaged for analysis.

### Angiotensin II infusions and intracerebroventricular liposome injections

Angiotensin II infusions and intracerebroventricular liposome injections were performed as described previously ^(20)^. Rats were anesthetized by an intraperitoneal injection of 50 mg/kg sodium pentobarbital. A small incision was made in the skin behind the neck, and the subcutaneous tissue was spread toward the interscapular space to create a pocket for an osmotic minipump (2002, Alzet, Cupertino, CA, USA), which was filled with either saline as a vehicle or angiotensin II (A9525, Sigma-Aldrich, St. Louis, MO, USA). The angiotensin II concentration was adjusted to an infusion rate of 150 ng/kg/min for 2 weeks. After implanting the osmotic minipump, rats were placed on a stereotaxic frame (Narishige Scientific Instruments, Tokyo, Japan) in a prone position. The skin overlying the midline of the skull was incised, and a small hole was drilled into the dorsal surface of the cranium 0.8 mm posterior to the Bregma and 1.5 mm lateral to the midline. The tip of a Hamilton syringe (10 μL) was lowered 4 mm vertically from the skull surface, and a total of 50 μL of either phosphate-buffered saline (PBS)-liposome or clodronate-liposome was injected bilaterally into the lateral ventricles, with a 20-minute injection time. The injection volume of clodronate-liposome was determined based on a previous report ^(18)^. Before and after surgery, each rat received a prophylactic intramuscular injection of 20,000 U/kg body weight of penicillin G. Postoperatively, all rats received a 2% salt diet until the end of the experiment. The following four groups were created: rats receiving a saline infusion with an intracerebroventricular injection of PBS-liposome (n = 5); rats receiving a saline infusion with an intracerebroventricular injection of clodronate-liposome (n = 5); rats receiving an angiotensin II infusion with an intracerebroventricular injection of PBS-liposome (n = 7); and rats receiving an angiotensin II infusion with an intracerebroventricular injection of clodronate-liposome (n = 6).

### Acute ganglionic blockade

Acute ganglionic blockade was performed as described previously ^(20)^. Different sets of rats (n = 22) were used to examine the effect of macrophage depletion in the CSF on the resting sympathetic activity of angiotensin II-salt-treated rats. Six rats received saline-salt treatment with an intracerebroventricular injection of PBS-liposome, and 16 rats received angiotensin II-salt treatments with an intracerebroventricular injection of either PBS-liposome (n = 8) or clodronate-liposome (n = 8). At 5-7 days after treatment initiation, the rats were anesthetized with sodium pentobarbital and underwent surgery for the implantation of arterial and venous catheters for BP recording and drug administration, respectively; they were then allowed to recover for 48 hours. At 7-9 days after treatment initiation, the acute ganglionic blockade experiment was performed in conscious, unrestrained rats. The rats were placed in an 18-cm diameter plastic bowl and allowed to move freely, with at least 30 minutes to adjust to the recording environment before BP recordings. The arterial pressure was recorded using the Power Lab system (ML750, ADI Instruments, Castle Hill, Australia). Resting arterial pressure and HR were recorded for at least 30 minutes. After recording resting BP, 40 mg/kg of hexamethonium, a ganglionic blocker, was administered intravenously to examine the rats’ resting sympathetic tone. The effect of acute ganglionic blockade on BP was monitored, and the degree of the peak depressor response was measured to evaluate resting sympathetic tone. Data were analyzed using the Chart program supplied by the Power Lab system.

### Real-time reverse transcription-polymerase chain reaction for inflammation-related cytokine expressions in the medulla oblongata of angiotensin II-salt-induced hypertensive rats

Real-time polymerase chain reaction (RT-PCR) was performed as described previously ^(19)^. At the end of the acute ganglionic blockade experiment, the deeply anesthetized rats were perfused with ice-cold PBS, and the rats’ brainstems were dissected and immersed in RNAlater solution (Thermo Fisher Scientific, MA, USA) overnight at 4°C, then stored at −80°C until future use. In 4-5 rats from each group, RNA was extracted from the brainstem homogenate using the RNeasy mini kit (Qiagen, Hilden, Germany) with DNase digestion according to the manufacturer’s instructions. Total RNA (0.5 μg) was reverse-transcribed to a single-stranded cDNA using High Capacity cDNA Reverse Transcription kits (Applied Biosystems, CA, USA). PCR was performed for inflammation-related cytokines on a Bio-Rad C1000 thermal cycler with a CFX96 detection system. Gene expressions of the following cytokines were examined using specific probes: Rn00579682_m1 for transforming growth factor-beta (TGF-β); Rn01525859_g1 for tumor necrosis factor-alpha (TNF-α); Rn01489669_m1 for interleukin (IL)-6; and Rn00563409_m1 for IL-10 (FAM/MGB probe, Applied Biosystems, CA, USA). The relative gene expressions were quantified using the comparative threshold cycle method with 18S (4319413E, VIC/MGB probe, Applied Biosystems, CA, USA) as a housekeeping gene reference.

### Histological examination

The fixation and immunostaining procedures were described previously ^(21)^. In brief, rats were perfused transcardially with 50 mL ice-cold PBS followed by 50 mL 4% paraformaldehyde in PBS. The brainstem was removed and post-fixed in a 4% paraformaldehyde solution for 1 hour and transferred to a phosphate buffer containing 20% sucrose at pH 7.4. The frozen brainstem was sectioned in the coronal plane at a thickness of 5 μm. The sections were incubated with tris(hydroxymethyl)aminomethane-ethylenediaminetetraacetic acid buffer (pH 9.0) at 98°C for 10 minutes for antigen retrieval. They were then incubated with antibodies against Iba1 (ab178846, Abcam, Cambridge, MA, USA; 1:200), followed by green-fluorescent dye-conjugated secondary antibodies (Alexa Fluor 488, Molecular Probes; 1:400). Sections incubated without the primary antibody were used as negative controls. The anatomical location of the rostral ventrolateral medulla (RVLM) within the section was determined using the Paxinos and Watson atlas. Iba1-positive cells were counted in each field (1.1 × 1.5 mm) including the RVLM using image analysis software (Image-Pro^Ⓡ^ Plus).

### Statistical analysis

The values are expressed as the mean ± standard error of the mean. Statistical differences among groups were assessed using analysis of variance (ANOVA), with or without repeated measures as appropriate. Post hoc comparisons to identify significant differences were conducted using Tukey’s Honestly Significant Difference test. p < 0.05 was considered statistically significant.

## Results

### Effect of macrophage depletion in the CSF on the time-dependent changes in the mean arterial pressure of angiotensin II-salt-induced hypertensive rats

**Figure 1.**
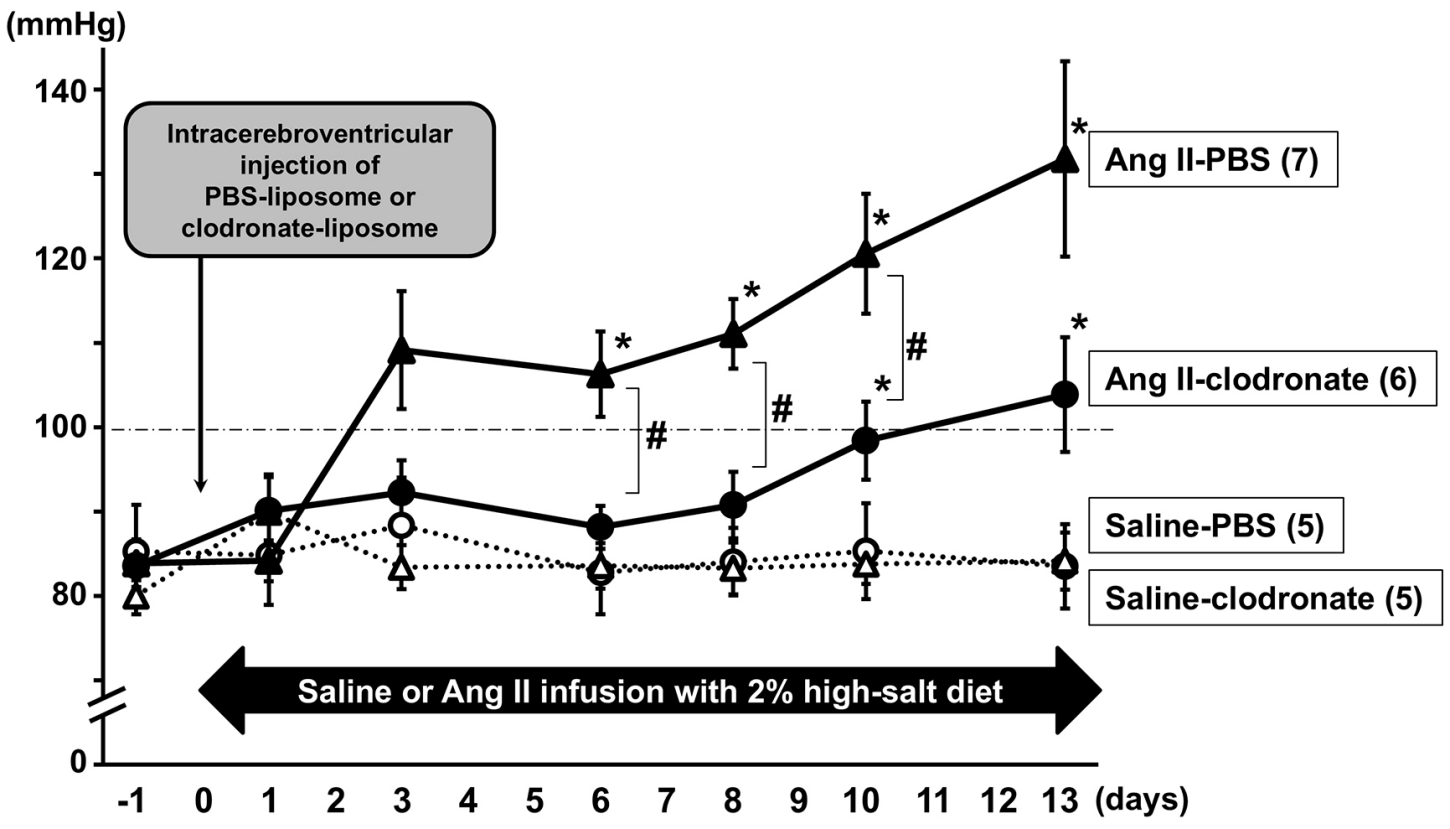
Time-dependent changes in the mean arterial pressure (MAP) of angiotensin II (Ang II)-salt-induced hypertensive rats. Male Sprague-Dawley rats with radio-telemetry pressure transducers underwent surgery for the subcutaneous implantation of either saline- or Ang II-filled osmotic minipump. They received an intracerebroventricular injection of either phosphate-buffered saline (PBS)- or clodronate-liposomes and were provided with a high-salt diet (2.0% NaCl). The saline-salt treatment groups are represented by a dotted line, with PBS-liposome (open triangle) or clodronate-liposome (open circle). The Ang II-salt treatment groups are represented by a solid line, with PBS-liposome (solid triangle) or clodronate-liposome (solid circle). The numbers in parentheses indicated the number of rats tested; *p < 0.05 versus the rats receiving saline-salt treatment with PBS-liposome or clodronate-liposome; #p < 0.05 versus the rats receiving Ang II-salt treatment with PBS-liposome.

To determine whether macrophages in the CSF are involved in the development of angiotensin II-salt-induced neurogenic hypertension, we intracerebroventricularly administered clodronate-liposomes to deplete macrophages in the CSF of angiotensin II-salt-treated rats and monitored their BP. Figure 1 shows the time-dependent changes in mean arterial pressure (MAP) of the rats implanted with a radio-telemetry pressure transducer. Repeated measures ANOVA revealed a significant difference in MAP between rats treated with angiotensin II-salt and PBS-liposomes and those receiving other treatments. The saline-salt treatment with intracerebroventricular injection of either PBS- or clodronate-liposome had a negligible effect on the MAP of rats during the experimental period. The MAP of rats receiving angiotensin II-salt treatment with PBS-liposome increased gradually and reached a significant increase at 6 days after the initiation of angiotensin II-salt treatment compared to that of rats receiving saline-salt treatments. Rats receiving intracerebroventricular injection of clodronate-liposome attenuated the angiotensin II-salt-induced MAP increases; however, a delayed increase in MAP was observed, and their MAP was significantly higher at 10 days after the initiation of angiotensin II-salt treatment compared to that of saline-salt-treated rats. Rats receiving angiotensin II-salt treatment with clodronate-liposome had significantly lower MAP than rats receiving angiotensin II-salt treatment with PBS-liposome at 6, 8, and 10 days after the initiation of angiotensin II-salt treatment (angiotensin II with PBS-liposome vs. angiotensin II with clodronate-liposome: 106 ± 5 vs. 89 ± 3 mmHg, 111 ± 4 vs. 91 ± 4 mmHg, 121 ± 7 vs. 101 ± 5 mmHg at 6, 8, and 10 days, respectively; p < 0.05). The HR did not differ among the groups during the experimental period.

### Effect of macrophage depletion in the CSF on the resting sympathetic activity of angiotensin II-salt-induced hypertensive rats

**Figure 2.**
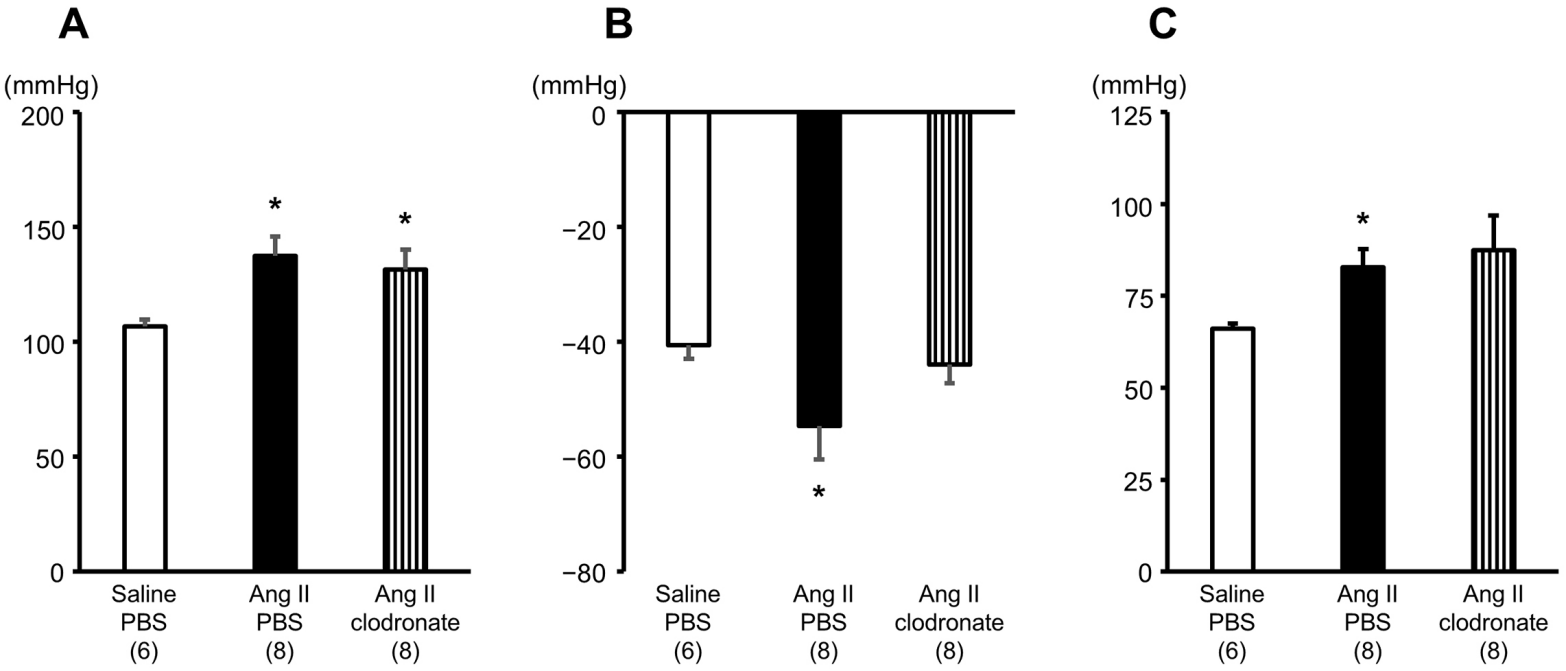
The depressor response to acute ganglionic blockade in angiotensin II (Ang II)-salt-induced hypertensive rats. A, Resting mean arterial pressure (MAP) of rats before the ganglionic blockade. Rats were treated with either saline or Ang II via subcutaneously implanted osmotic minipumps and received an intracerebroventricular injection of phosphate-buffered saline (PBS)-liposome or clodronate-liposome. B, Peak depressor response to acute ganglionic blockade. Following the same treatment paradigm, ganglionic blockade was performed to evaluate peak MAP reductions. C, MAP measured after ganglionic blockade. Post-blockade MAP was recorded in the same groups of rats. The numbers in parentheses indicate the number of rats tested; *p < 0.05 versus the rats receiving saline-salt treatment with an intracerebroventricular injection of PBS-liposome.

Figure 2 shows the results of acute ganglionic blockade performed in rats at 7-9 days after the initiation of angiotensin II-salt treatment. We chose this evaluation timing because a difference in resting MAPs was observed between rats receiving angiotensin II-salt treatment with PBS-liposome and those receiving angiotensin II-salt treatment with clodronate-liposome in the telemetry experiment. Figure 2A shows the resting MAPs before ganglionic blockade. The angiotensin II-salt treatment significantly increased the resting MAP of rats receiving intracerebroventricular injection of either PBS-liposome (137 ± 6 mmHg) or clodronate-liposome (131 ± 9 mmHg) compared to the MAPs of rats receiving saline-salt treatment with PBS-liposome (107 ± 3 mmHg). The angiotensin II-salt treatment significantly increased the peak depressor response to acute ganglionic blockade in rats receiving intracerebroventricular injection of PBS-liposome (−54 ± 6 mmHg) compared to rats receiving saline-salt treatment with PBS-liposome (−41 ± 2 mmHg). Intracerebroventricular injection of clodronate-liposome in angiotensin II-salt-treated rats did not significantly alter the peak depressor response (−44 ± 3 mmHg) compared to saline-salt treatment with PBS-liposome. No significant difference was observed between angiotensin II-salt-treated rats receiving PBS- or clodronate-liposomes (Figure 2B). The MAP attained after the ganglionic blockade was higher in rats receiving angiotensin II-salt treatment with PBS-liposome (83 ± 5 mmHg) than in rats receiving saline-salt treatment with PBS-liposome (66 ± 4 mmHg). Rats receiving angiotensin II-salt treatment with clodronate-liposome appeared to have higher MAPs attained after the ganglionic blockade; however, their MAPs varied considerably (87 ± 9 mmHg) (Figure 2C).

### Expression of inflammation-related cytokines in the medulla oblongata of angiotensin II-salt-induced hypertensive rats

**Figure 3.**
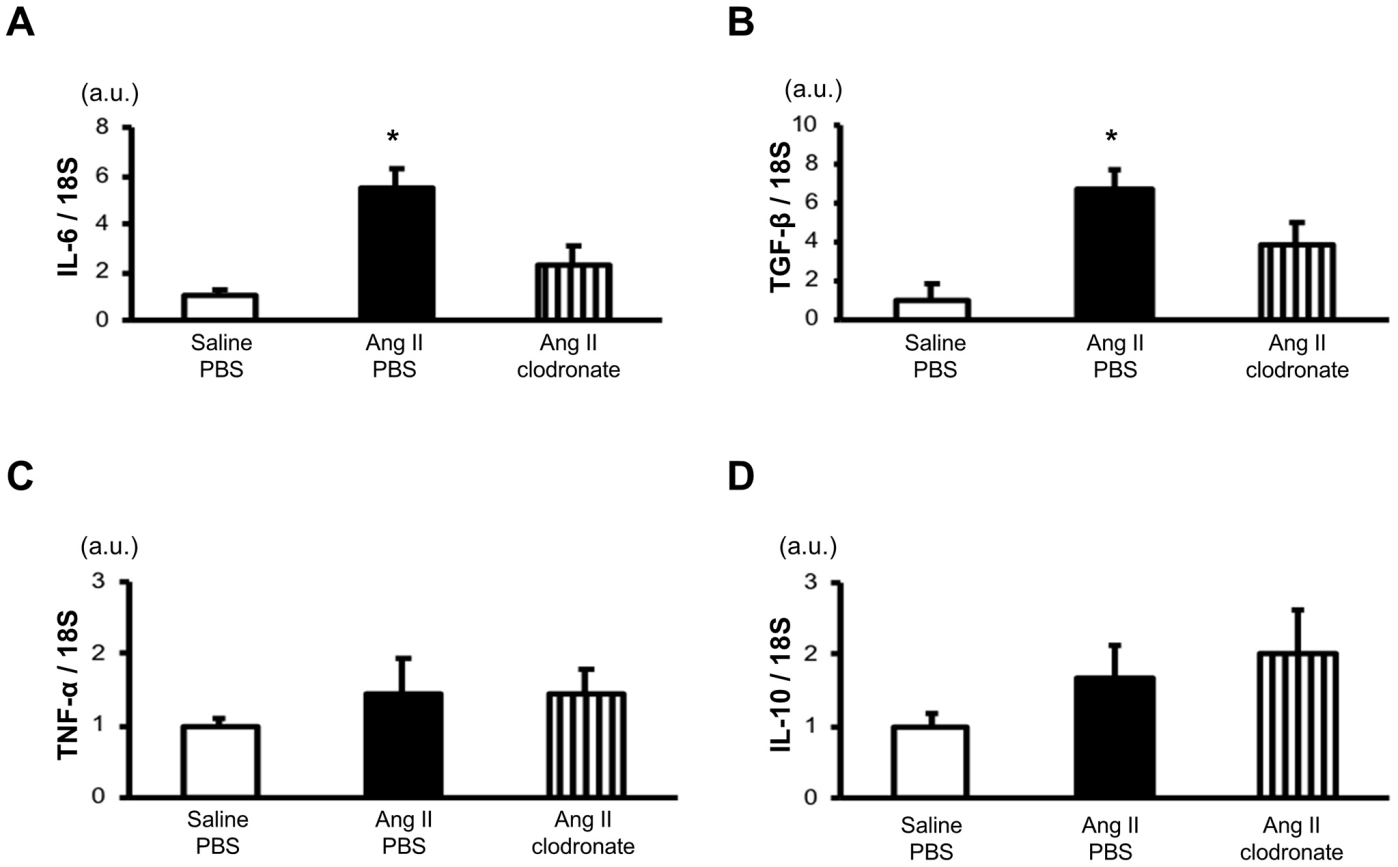
mRNA levels of inflammatory cytokines in the medulla oblongata of angiotensin II (Ang II)-salt-induced hypertensive rats. Total RNA extracted from the homogenates of the medulla oblongata was analyzed by Real-time reverse transcription-polymerase chain reaction (RT-PCR) to assess the mRNA expression of interleukin (IL)-6 (A), transforming growth factor-beta (TGF-β) (B), tumor necrosis factor-alpha (TNF-α) (C), and IL-10 (D). Rats were treated with either saline or Ang II via subcutaneously implanted osmotic minipumps and received an intracerebroventricular injection of phosphate-buffered saline (PBS)-liposome or clodronate-liposome. Values are expressed as fold changes relative to the saline-salt treatment group with PBS-liposome. *p < 0.05 versus rats receiving saline-salt treatment with PBS-liposome; n = 4-5 animals per group. a.u.: arbitrary unit.

Figure 3 shows the mRNA expression of inflammatory cytokines in the medulla oblongata of angiotensin II-salt-treated rats. Rats receiving angiotensin II-salt treatment with PBS-liposome had significantly higher IL-6 and TGF-β mRNA expressions (5.5 and 6.8 times, respectively) in the medulla oblongata than rats receiving saline-salt treatment with PBS-liposome. The depletion of macrophages by the intracerebroventricular injection of clodronate-liposome attenuated the angiotensin II-salt-mediated increase in IL-6 and TGF-β mRNA expressions. The angiotensin II-salt treatment or intracerebroventricular injection of clodronate-liposome did not affect TNF-α and IL-10 mRNA expressions in the medulla oblongata.

### The number of microglial cells in the RVLM of angiotensin II-salt-induced hypertensive rats

**Figure 4.**
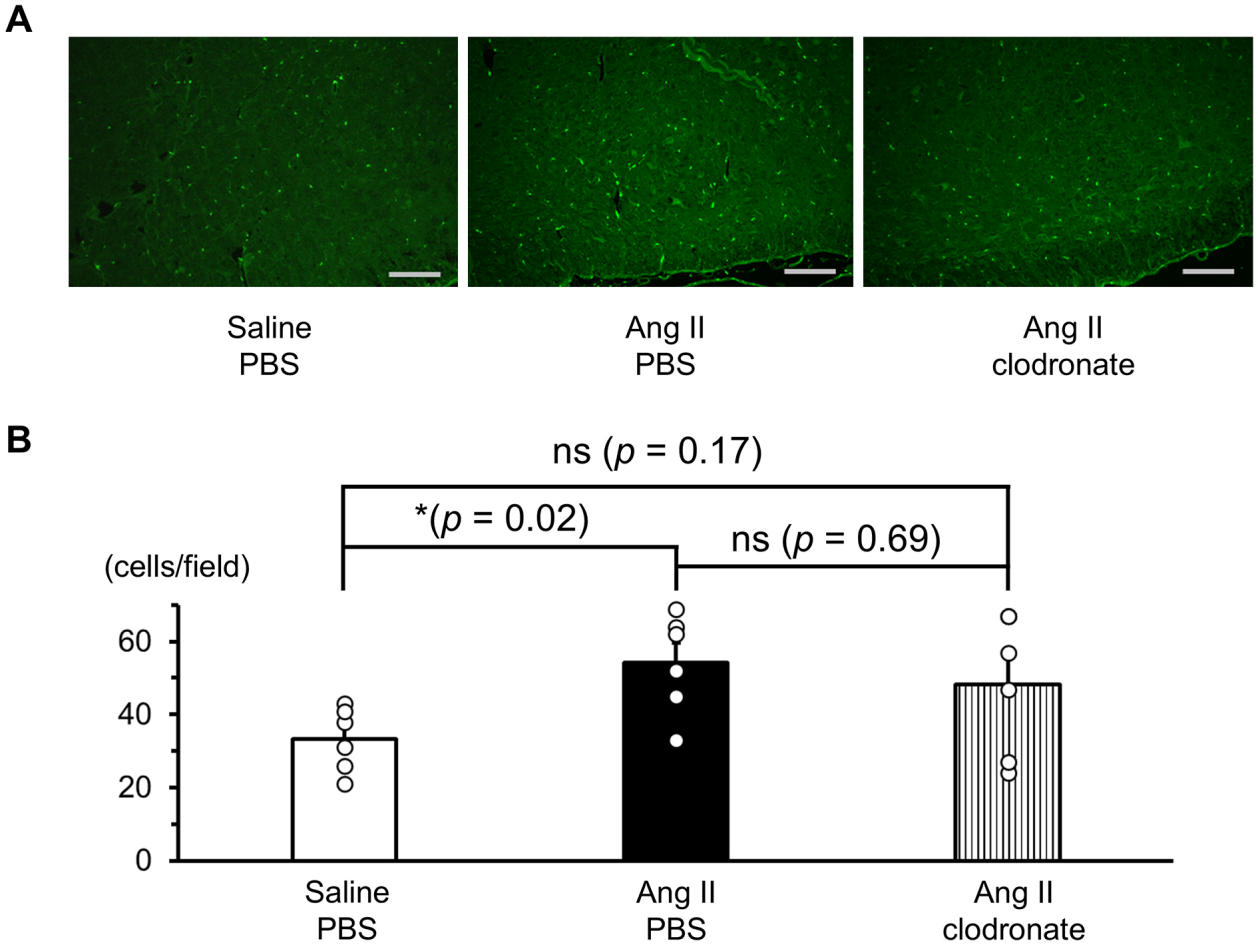
Immunoreactivity of Iba1 in the rostral ventrolateral medulla (RVLM) of angiotensin II (Ang II)-salt-induced hypertensive rats. A, Representative photo of Iba1 immunoreactivity in the RVLM of rats. The scale bar denotes 200 μm. B, The number of Iba1 positive cells in the RVLM. Rats were treated with either saline or Ang II via subcutaneously implanted osmotic minipumps and received an intracerebroventricular injection of PBS-liposome or clodronate-liposome. Iba1-positive cells were counted in two microscopic fields per rat. Individual data points are overlaid on the bar graphs. In the Ang II-salt treatment group receiving clodronate-liposomes, two data points overlapped at a value of 67. Data are presented as the mean ± standard error of the mean; n = 3 animals per group. n = 3 animals per group, two fields per rat were examined; *p < 0.05 versus the rats receiving saline-salt treatment with PBS-liposome. ns: not significant.

Figure 4A shows a representative image of the Iba1, a marker of microglia, immunoreactivity in the RVLM. The image covers a region broader than the RVLM to improve anatomical orientation and clarity. The number of Iba1-positive cells in the RVLM was significantly increased in rats receiving angiotensin II-salt treatment compared to rats receiving saline-salt treatment. However, rats receiving angiotensin II-salt treatment with intracerebroventricular injection of clodronate-liposome did not show a significant increase in the number of Iba1-positive cells in the RVLM compared to rats receiving saline-salt treatment (Figure 4B).

## Discussion

**Figure 5.**
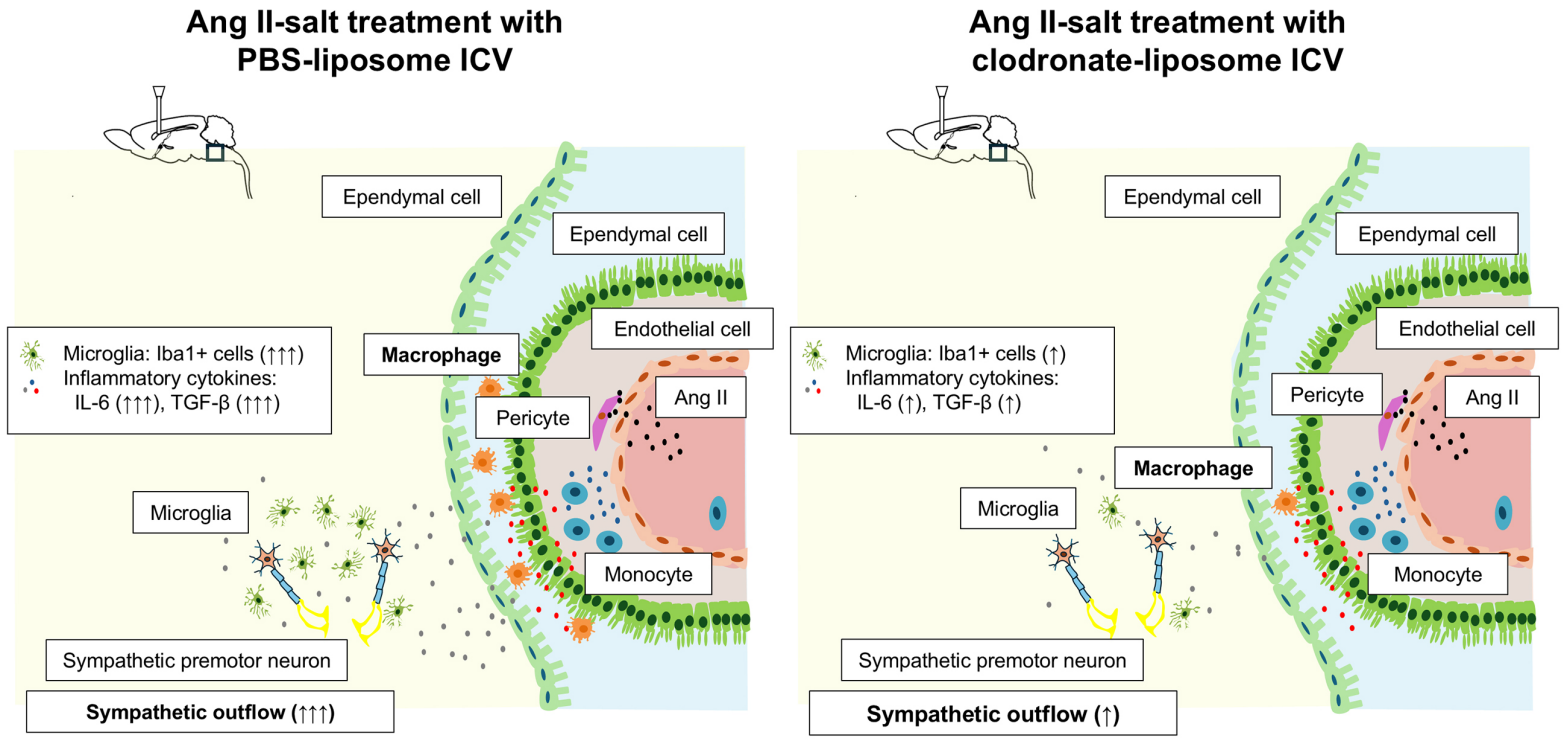
Hypothetical illustration of cardiovascular regulatory areas in the brain. These illustrations highlight cardiovascular regulatory regions in the brain and present our working hypothesis on the role of cerebrospinal fluid (CSF) macrophages in angiotensin II (Ang II)-induced neurogenic hypertension. CSF macrophages may relay inflammatory signals from peripheral Ang II to the brain parenchyma, promoting sympathetic overactivity via inflammation in these regulatory areas (left). Macrophage depletion via intracerebroventricular clodronate-liposome administration attenuates the development of Ang II-salt-induced neurogenic hypertension in rats (right).

This study demonstrated that the intracerebroventricular administration of clodronate-liposomes to deplete the macrophages in the CSF of angiotensin II-salt-treated rats attenuated a time-dependent increase in arterial BP. The intracerebroventricular administration of clodronate-liposomes also attenuated the increase in the peak depressor response to intravenous hexamethonium injection, mRNA expression of IL-6 and TGF-β in the brainstem of angiotensin II-salt-treated rats. Our results suggest that macrophages in the CSF may play a role in the development of angiotensin II-salt-induced neurogenic hypertension by modulating brain inflammation (Figure 5).

Macrophages may play a role in the increase in BP among hypertensive animals. A single intravenous injection of clodronate-liposomes to deplete circulating macrophages attenuated the increase in MAP in DOCA-salt-treated rats ^(22)^. Furthermore, the intravenous injection of clodronate-liposomes attenuated the increase in systolic BP in Dahl salt-sensitive rats ^(23)^. These results suggest that circulating macrophages may be involved in the development of hypertension. Additionally, in a study using stroke-prone spontaneously hypertensive rats (SHRSP), the intracerebroventricular administration of clodronate-liposomes attenuated the increase in MAP and reduced the depressor responses to intravenous hexamethonium injections ^(18)^. This suggests that macrophages in the CSF may be involved in the neurogenic mechanisms, including sympathetic overactivation, that increase BP in a genetic model of severe hypertension and cerebral stroke. In the present study, rats receiving angiotensin II-salt treatment with a single intracerebroventricular administration of clodronate-liposomes had significantly lower MAP than rats receiving angiotensin II-salt treatment with intracerebroventricular administration of PBS-liposomes at 6, 8, and 10 days after the initiation of angiotensin II-salt treatment in the telemetry experiment (Figure 1). The intracerebroventricular injection of clodronate-liposomes in angiotensin II-salt-treated rats attenuated the peak depressor responses to acute ganglionic blockade (Figure 2B). These results suggest that macrophages in the CSF may be involved in the development of angiotensin II-salt-induced neurogenic hypertension.

The mechanism by which macrophages in the CSF activate the sympathetic nervous system and elevate BP is not yet fully understood. Previous studies have suggested that neurogenic hypertension induced by a peripheral infusion of angiotensin II may involve the inflammation of cardiovascular regulatory areas in the brain. In Sprague-Dawley rats, a chronic subcutaneous infusion of angiotensin II caused an increase in MAP, plasma norepinephrine levels, the number of activated microglial cells in the paraventricular nucleus (PVN), and mRNA levels of inflammatory cytokines in the hypothalamus, including the PVN ^(10), (24)^. Furthermore, Sprague-Dawley rats receiving a continuous subcutaneous infusion of angiotensin II with a single intracerebroventricular administration of autologous mesenchymal stem cells showed attenuation in sympathetic overactivation and BP elevation, which may have modulated inflammation in cardiovascular regulatory areas of the brain ^(20)^. Additionally, in Sprague-Dawley rats receiving an intraperitoneal infusion of lipopolysaccharide, an animal model of peripheral inflammation, MAP was elevated, and the expression of Iba-1 protein and distribution of Iba-1 immunoreactivity in the RVLM were markedly increased ^(25)^. The studies mentioned above suggest that the increase in peripheral inflammatory signals, including those from angiotensin II, induces inflammation in the cardiovascular regulatory areas of the brain and causes neurogenic hypertension. However, it remains unclear how peripheral inflammatory signals transmit inflammation to the brain parenchyma, given the well-known barriers, such as the blood-brain barrier and blood-CSF barrier. In a previous experimental study using mice, angiotensin II type 1 receptors in perivascular macrophages were reported to be possibly involved in angiotensin II-induced blood-brain barrier dysfunction ^(11)^. Continuous subcutaneous administration of angiotensin II leads to increased permeability of the blood-brain barrier in mice. In contrast, in mice where the angiotensin II type 1a receptor was knocked down in perivascular macrophages, continuous administration of angiotensin II did not significantly alter blood-brain barrier permeability. In a study using SHRSP, intraventricular injection of clodronate-liposomes to deplete perivascular macrophages reduced the increase in mean BP and attenuated the depressor effect of hexamethonium. Immunostaining for c-Fos, a marker of neuronal activation, showed a decrease in this marker in the PVN and RVLM regions. Additionally, immunostaining results showed a decreased number of cluster of differentiation 163+/ prostaglandin E2+ and cluster of differentiation 163-positive cyclooxygenase-2 cells ^(18)^. In the present study, the angiotensin II-salt-treated rats showed increased mRNA expressions of IL-6 and TGF-β in the medulla oblongata and increased numbers of Iba1-positive cells in the RVLM (Figure 3A and B, 4A and B). The depletion of macrophages by intracerebroventricular injection of clodronate-liposomes attenuated the angiotensin II-salt-mediated increase in IL-6 and TGF-β mRNA expressions in the medulla oblongata in the RVLM of angiotensin II-salt-treated rats. Taken together, these findings suggest that macrophages in the CSF may play a role in transmitting the inflammatory signals of peripheral angiotensin II to the brain parenchyma and contribute to sympathetic nerve hyperactivity through the induction of brain inflammation in the cardiovascular regulatory areas by activated inflammatory microglial cells (Figure 5).

The present study has several limitations. First, the attenuation of BP elevation by a single intraventricular administration of clodronate-liposomes to deplete macrophages in the CSF was transient. The reasons for this observation may include the transient effect of clodronate-liposomes or the potential migration of circulating macrophages into the CSF. The outcome of continuous intracerebroventricular administration of clodronate-liposomes to deplete macrophages in the CSF remains unclear. Second, it was not possible to identify the source of the macrophages. However, this study did not aim to identify the source of the macrophages, as inflammatory conditions are known to increase the presence of peripheral blood-derived macrophages in the CSF ^(26)^. Therefore, we assume that the intracerebroventricular administration of clodronate-liposomes primarily depleted CSF-specific macrophages but also circulating macrophages in the CSF. Third, it remains unclear whether the intracerebroventricular injection of clodronate-liposomes reached the brain parenchyma and directly affected microglia. Previous studies have reported that clodronate-liposomes deplete microglia ^(27)^, and this issue should be addressed in future research. Fourth, we analyzed only MAP; systolic and diastolic pressures were not analyzed. Occasional signal dampening, likely from thrombus at the catheter tip or vessel wall contact, reduced pulse pressure. Fifth, we did not conduct experiments involving acute ganglionic blockade, RT-PCR for inflammation-related cytokine expressions in the medulla oblongata, or histological examination of the RVLM in the group of rats receiving a saline infusion with intracerebroventricular injection of clodronate-liposomes. Sixth, sympathetic tone was not directly measured but inferred via acute ganglionic blockade with hexamethonium. Seventh, only mRNA of inflammatory cytokines was measured; protein levels were not. Thus, mRNA may not reflect protein expression due to post-transcriptional regulation. Eighth, we conducted only RVLM histological examination. The examination of other cardiovascular regulatory areas in the brain is an important topic. In our study, the detailed mechanisms of how brain inflammation induces sympathetic overactivation in cardiovascular regulatory areas remain unclear. Finally, it remains unclear whether the depletion of macrophages in the CSF would attenuate the elevated BP in the established phase of neurogenic hypertension. These limitations represent important topics for future research. It is considered a clinical challenge to explore therapeutic approaches aimed at managing neurogenic hypertension by partially inhibiting the function of macrophages through intracerebroventricular methods.

In conclusion, our results suggest that macrophages in the CSF may be involved in the development of angiotensin II-salt-induced neurogenic hypertension by modulating brain inflammation. The development of a treatment modality for neurogenic hypertension based on the mechanism of macrophages in the CSF is a subject for future investigation.

## Article Information

### Acknowledgments

This paper is based on experimental results obtained by the authors during their affiliation with the Department of Cardiovascular Medicine, Nephrology and Neurology, Graduate School of Medicine, University of the Ryukyus. We thank Sakiko Hatoma-Kina, Morihiro Ota, and Masato Nohara for their excellent technical assistance.

### Author Contributions

Conceptualization, Masanobu Yamazato; Methodology, Masanobu Yamazato, Yoriko Yamazato; Formal analysis, Masanobu Yamazato; Investigation, Masanobu Yamazato, Yoriko Yamazato; Resources, Masanobu Yamazato; Data curation, Masanobu Yamazato; Writing―original draft preparation, Yoshitsugu Sunagawa, Masanobu Yamazato; Writing―review and editing, Yoshitsugu Sunagawa, Masanobu Yamazato, Yoriko Yamazato, Akio Ishida, Takuto Nakamura, and Yusuke Ohya; Visualization, Yoshitsugu Sunagawa, Masanobu Yamazato; Supervision, Yusuke Ohya; Project administration, Masanobu Yamazato; Funding acquisition, Masanobu Yamazato. All authors have read and agreed to the published version of the manuscript.

### Conflicts of Interest

None

### Institutional Review Board Approval

The experimental protocol was approved by the Animal Care and Use Committee at the University of the Ryukyus (A2017155, A2021011).
